# Antioxidant Properties of Selected Flavonoids in Binary Mixtures—Considerations on Myricetin, Kaempferol and Quercetin

**DOI:** 10.3390/ijms241210070

**Published:** 2023-06-13

**Authors:** Małgorzata Olszowy-Tomczyk, Dorota Wianowska

**Affiliations:** Department of Chromatography, Institute of Chemical Sciences, Faculty of Chemistry, Maria Curie-Skłodowska University in Lublin, Pl. Maria Curie-Skłodowska 3, 20-031 Lublin, Poland; malgorzata.olszowy-tomczyk@mail.umcs.pl

**Keywords:** flavonoids, antioxidant activity, binary mixture, antagonistic antioxidant effect, additive antioxidant effect, synergistic antioxidant effect

## Abstract

Flavonoids, secondary plant metabolites with many health-promoting properties, including antioxidant, are a valuable component of food products, especially functional foods. In the latter, plant extracts are commonly used, the properties of which are attributed to the characteristic main ingredients. However, in a mixture the antioxidant properties of the individual ingredients do not always show an additive effect. This paper presents and discusses the antioxidant properties of naturally occurring flavonoid aglycones and their binary mixtures. In the experiments, model systems were used that differed in the volume of the alcoholic antioxidant solution in the measuring system and its concentration in the range in which it occurs in nature. Antioxidant properties were determined by ABTS and DPPH methods. The presented data proved that the dominant resultant effect in the mixtures is antioxidant antagonism. The magnitude of the observed antagonism depends on the mutual relations of individual components, their concentrations and the method used to assess antioxidant properties. It was shown that the observed non-additive antioxidant effect of the mixture results from the formation of intramolecular hydrogen bonds between phenolic groups of the antioxidant molecule. The presented results may be useful in the context of proper design of functional foods.

## 1. Introduction

The recently observed increase in awareness of food’s impact on human health means that more and more importance is attached to a well-considered choice of consumed food products. This is the result of the warnings of doctors who, in their daily practice, see the relationship between an improper diet and the occurrence of civilization diseases and aging at the molecular level, as well as deliberate education of the society in recognizing the nutritional value of individual food products [1,2]. With these two aspects in mind, the concept of the so-called functional foods was created [3].

When designing functional food, special attention is paid to the use of natural ingredients with antioxidant properties [4]. These compounds are assigned a double role in the product [5]. They are to protect the food item against adverse changes resulting from its aging process, and bearing in mind that natural compounds are considered safe, a product with natural preservatives is better (healthier) than a product with artificial preservatives. More importantly, natural antioxidants from functional foods are also supposed to supplement the human body with health-promoting substances. This is the basic and easiest way to protect the body against the harmful effects of reactive oxygen species, including free radicals that are the activators of many dangerous diseases (including diabetes, hypertension, atherosclerosis and cancer) [6,7].

**Figure 1:**
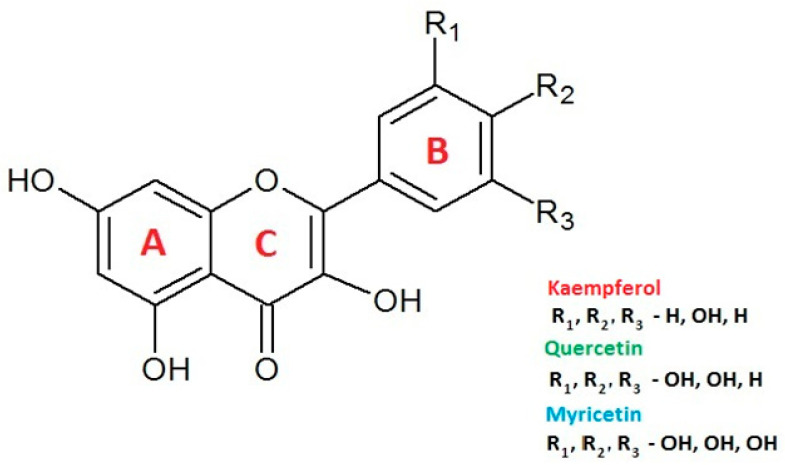
Chemical structures of the examined compounds.

Plant extracts, especially those rich in polyphenolic compounds, are the source of natural antioxidants commonly used in the creation of functional foods. Myricetin, quercetin and kaempferol are representatives of one of their subgroups, i.e., flavonols, which, due to their high prevalence in the plant kingdom, are an important component of a healthy diet [8,9,10,11]. The structures of these compounds are shown in Figure 1. They are characterized by the presence of a C6-C3-C6 skeleton forming the A-C-B ring system, with one, two and three hydroxyl groups present in the B ring of kaempferol, quercetin and myricetin, respectively.

Phenolic compounds exhibit various antioxidant properties including preventing the formation of reactive species, neutralizing (scavenging) free radicals, forming chelate complexes with pro-oxidizing metals and removing or repairing damage caused by reactive compounds [12]. In mixtures, the antioxidant properties of individual components contribute to the resultant antioxidant capacity of the extract, and according to the literature [13], these properties do not always show an additive effect. A synergistic or antagonistic mutual interaction of antioxidants on each other is equally likely [14,15]. In the former, the resultant antioxidant effect is higher than the sum of the individual effects. In the second, the resultant effect is lower than the sum of the individual effects.

There are various reasons for the antagonistic (synergistic) effects of antioxidants in mixtures. The most commonly reported is the regeneration of a less effective (more effective) antioxidant by a more (less) effective antioxidant [13,16,17]; the formation of stable intermolecular complexes (dimers and adducts) between the tested antioxidants, which show weaker (greater) antioxidant activity than the parent antioxidants [15], and the difference in the reaction kinetics between a given antioxidant and a neutralized radical [15].

In the literature, one can find many articles on the study of the antioxidant properties of phenolic compounds. There are also papers on synergistic and antagonistic effects of antioxidant activity in mixtures of compounds, including flavonoids [18]. Nevertheless, given the number of known flavonoids, the very complex nature of the antioxidant effect and the impossibility of predicting the resultant antioxidant activity of the mixture, it is necessary to conduct further research to supplement and extend the knowledge about the antioxidant properties of compounds in mixtures as there is still room to show a new aspect of this issue even in a seemingly well-established area of research.

The literature emphasizes that the observed resultant antioxidant effect of the mixture depends on the type of antioxidant or the research method used. Antioxidant activity is typically determined for a particular concentration ratio of selected antioxidants. The impact of changing the concentration ratio on the observed effect of the resultant action of the mixture is rarely determined. Therefore, the research presented in this paper fills this gap by discussing the concentration-dependent formation of intramolecular hydrogen bonds and their effect on the resultant antioxidant activity of the mixture. Myricetin, quercetin and kaempferol, i.e., “healthy flavonoid compounds” that are aglycone forms of flavonols ubiquitous in nature and assimilable by the human body, were selected for the study. The experiments took into account the effect of both different concentrations of antioxidants in the range in which these compounds are typically found in plants and different volume ratios of components in the measurement system. Two spectrophotometric methods for measuring color radicals (ABTS^●+^ and DPPH^●^) were used. These methods seem to be the most popular and willingly used due to the simplicity of measurement, short time of conducting research and the use of an inexpensive and widely available spectrophotometer. It should be added that despite the identical methodology for measuring antioxidant properties, these tests are not the same. The model DPPH and ABTS radicals differ structurally, which may differentiate the structure–activity relationship of the antioxidant in both methods [19].

## 2. Results

**Figure 2:**
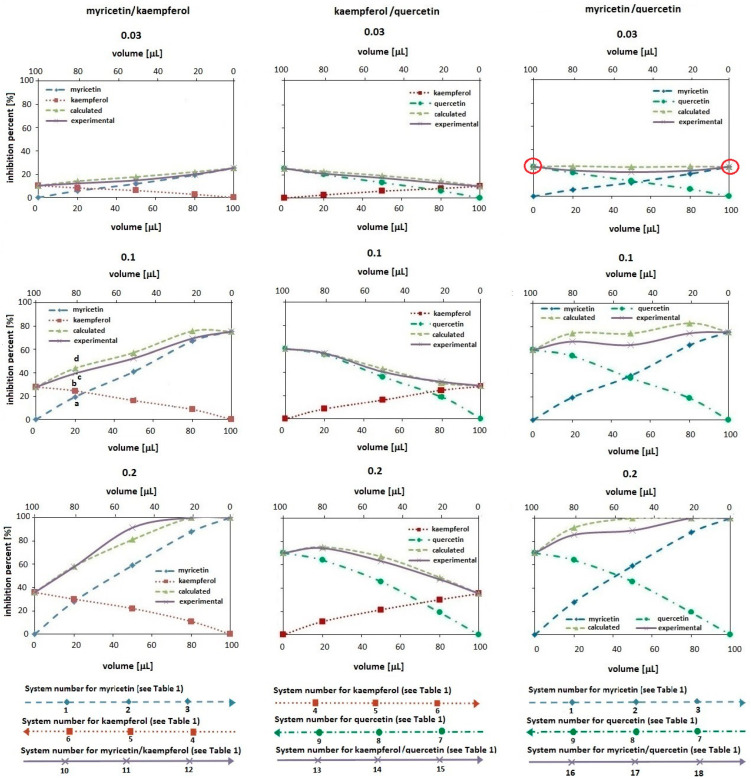
The antioxidant activity changes assessed by the ABTS method for the systems containing different volumes of myricetin solution (dashed line with diamonds), kaempferol solution (dotted line with squares), quercetin solution (dashed–dotted line with circles) and their binary mixtures (solid line with “x”) (see Table 1). The dashed line with triangles corresponds to the expected activity values for the tested pairs of compounds. The experimental values are the mean values for *n* = 5.

**Figure 3:**
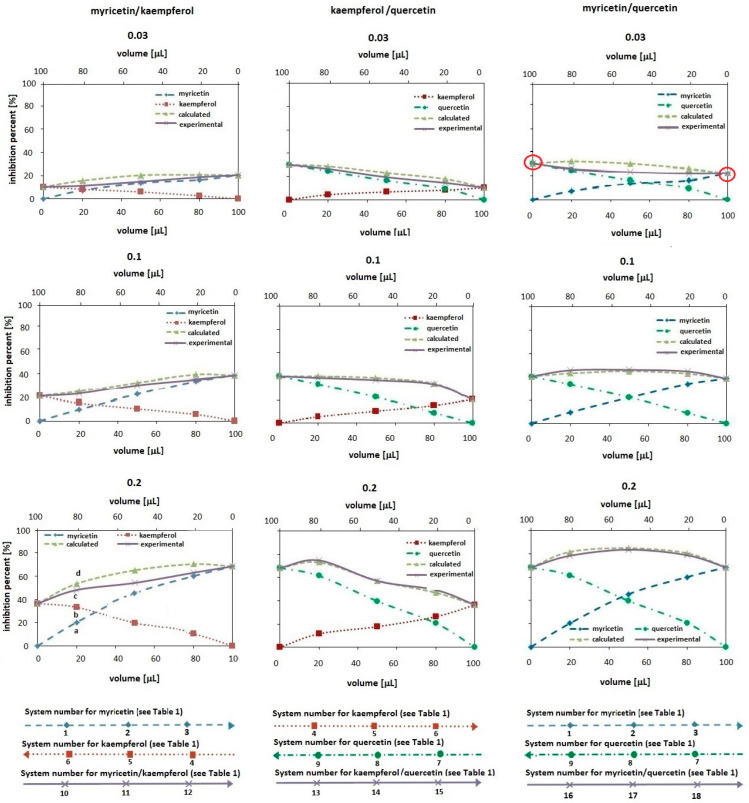
The antioxidant activity changes assessed by the DPPH method for the systems containing different volumes of myricetin solution (dashed line with diamonds), kaempferol solution (dotted line with squares), quercetin solution (dashed–dotted line with circles) and their binary mixtures (solid line with “x”) (see Table 1). The dashed line with triangles corresponds to the expected activity values for the tested pairs of compounds. The experimental values are the mean values for *n* = 5.

Figure 2 and Figure 3 present the influence of kaempferol or myricetin or quercetin amount in one-component solutions and in their binary mixtures on the antioxidant activity of the measuring system. The studies used methanolic solutions of the tested compounds at three concentration levels, i.e., 0.03, 0.1 and 0.2 mmol/L, which were mixed in three volume ratios: 20/80, 50/50 and 80/20 v/v (see Table 1) to obtain three binary mixtures: myricetin/kaempferol, kaempferol/quercetin and myricetin/quercetin. To make it easier to relate individual experimental points in the graphs with the sample numbers given in Table 1, three axes are provided at the bottom of each figure.

Figure 2 shows the results obtained by the ABTS method, whereas Figure 3 presents data which were estimated by the DPPH method. In the figures, the dashed line with diamonds represents the antioxidant activity changes for the systems containing different volumes of myricetin methanolic solution, the dotted line with squares shows the changes for the systems with different volumes of kaempferol methanolic solution and the dashed–dotted line with circles presents the changes for the systems that differ in volumes of quercetin methanolic solution. There are also two additional lines in each of the plots: the solid line with "x" shows the antioxidant activity changes for the experimental systems containing different volumes of the test compounds in the binary mixtures (the experimental curve in short), and the dashed line with triangles presents the calculated value of the antioxidant activity in the mixtures (the calculated curve in short). The latter is constructed by summing up the experimental inhibition percent obtained for the individual antioxidants forming the binary mixture. In the plots the antioxidant amounts in the measuring system are expressed as the volume of its solution for a given concentration in a 100 μL sample introduced to the system. The solution volume of the first component in the pair, e.g., the myricetin volume in the myricetin/kaempferol pair, is shown on the lower X axis. In turn, the volume of the second component in the same pair, i.e., kaempferol, is shown on the upper X axis. In the figures, the way of assigning the axes is shown with arrows at the bottom. Note that the values change inversely on both axes (the values on the top axis should be viewed from right to left). 

For a better understanding of the presented data, for the pair of antioxidants myricetin–kaempferol with a concentration of 0.1 mmol/L in the ABTS method (see Figure 2) and a concentration of 0.2 mmol/L in the DPPH method (see Figure 3), four points (a, b, c and d) were marked in the figures:

- Point “a” refers to the inhibition percent in the measuring system containing 20 μL of myricetin methanolic solution and 80 μL of MeOH in a 100 μL sample (system no 1 in Table 1);
- Point “b” refers to the inhibition percent in the system containing 80 μL of kaempferol methanolic solution and 20 μL of MeOH in a 100 μL sample (system no 6 in Table 1);
- Point “c” refers to the inhibition percent in the system containing 20 μL of myricetin and 80 μL of kaempferol (both dissolved in MeOH) (system no 10);
- Point “d” is the so-called “theoretical” point representing the inhibition percent expected for the mixture containing 20 μL of myricetin and 80 μL of kaempferol, assuming the additive antioxidant effects. Hence, the value at point “d” was calculated by summing up the inhibition percent obtained for the sample contained 20 μL of myricetin with 80 μL of MeOH (the value at point “a”) and 80 μL of kaempferol with 20 μL of MeOH (the value at point “b”).

The results of the statistical analysis of differences between the experimentally determined value of the antioxidant activity of a given pair of flavonoids and the value calculated as a result of summing up the activity of individual compounds forming a given pair for different volume ratios of methanolic antioxidant solutions at the three concentration levels are presented in Table 2 and Table 3 for the ABTS and DPPH methods, respectively. It was assumed in the considerations that the lack of a statistically significant difference between these values confirms the additive nature of the antioxidant activity in the binary mixture. Statistically significant differences in the results, in turn, confirm the non-additive nature of the antioxidant effect, with a negative value of this difference indicating the antagonistic behavior of the compounds in the binary mixture and a positive value indicating the synergistic effect. The results of these considerations are also presented in Table 2 and Table 3. In the tables, “I_e_” is the inhibition percent of the binary mixture determined experimentally, and “I_c_” is the calculated inhibition percent.

## 3. Discussion

### 3.1. Antioxidant Activity of Single-Component Solutions

As can be seen from the plots presented in Figure 2 and Figure 3, regardless of the method used to assess the antioxidant properties and the type of antioxidant, the curves reflecting the change in the antioxidant properties of one-component solutions show an increase in the inhibition percent with the increase in the volume of the methanolic antioxidant solution in the measuring system. This effect is greater the greater the concentration of a given antioxidant (compare the curves for 0.03 and 0.2 mmol/L). In addition, this effect depends on the type of antioxidant (its structure).

As is known from the literature [20], the greater the number of hydroxyl substituents present in the B ring, the lower the redox potential and the stronger the antioxidant properties. The results presented in Figure 2 and Figure 3 confirm this information because, among the tested compounds, myricetin has the greatest antioxidant activity (three hydroxyl groups per molecule in the B ring) and kaempferol has the lowest (one hydroxyl group per molecule). In the same order, the value of the redox potential changes from 0.3 V for myricetin, through 0.37 V for quercetin to 0.46 V for kaempferol. Thus, the increase in the value of the redox potential in the studied group of flavonoids actually reflects the decrease in their antioxidant properties. An exception to this rule, however, are the data presented for solutions of myricetin and quercetin at the lowest concentration, i.e., 0.03 mmol/L. A comparison of the position of the curves (marked with circles in the graphs) shows that, depending on the method, both solutions show either similar antioxidant properties (in the ABTS method) or weaker for the myricetin solution (in the DPPH method). This fact sheds new light on the knowledge about the antioxidant properties of single-component solutions.

In the case of polyphenolic compounds, not only the number of hydroxyl groups differentiates the antioxidant properties of individual compounds. Their location should also be taken into account. Flavonoids show better radical scavenging and/or antioxidant activity when their molecules contain hydroxyl substituents in the ortho position of the B ring [21]. Hence, the lack of a diphenolic structure in the B ring of the kaempferol molecule may explain the worst antioxidant activity of this compound. Nevertheless, another factor differentiating the antioxidant properties of individual components is their ability/susceptibility to form intramolecular hydrogen bonds. According to Leopoldini et al. [22], hydrogen bond interactions are necessary to increase the stability of antioxidant radicals; therefore, catechol (diphenolic) functionality is the main feature affecting the antioxidant activity. The radical formation from the hydroxyl group in catechol leads to species where the electron appears to be delocalized throughout the molecule, which may reduce the antioxidant properties of the compound [12]. 

**Figure 4:**
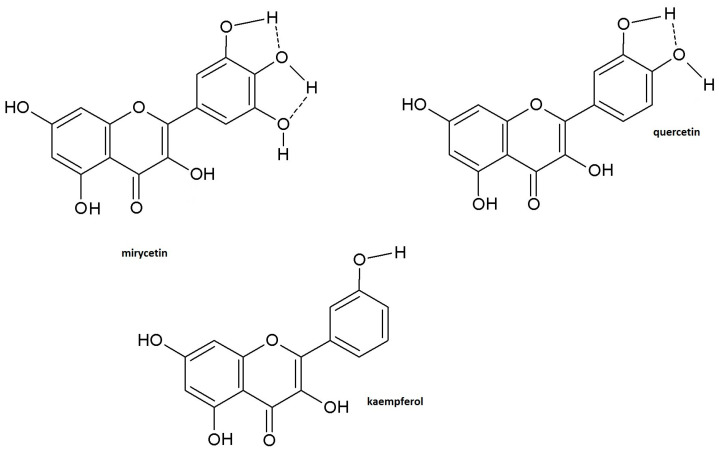
The chemical structures of flavonoids under study with indicated redox-active moieties and the possible sites of intramolecular H-bond formation.

As shown in Figure 4, the stabilization of the semiquinone radical formed in the first stage of oxidation of hydroxyl groups in the B ring of flavonols can be carried out in several ways. One of them is the formation of hydrogen bonds with neighboring OH groups. Especially in myricetin, the presence of three hydroxyl groups may further enhance the stabilizing effect due to its greater ability to form hydrogen bonds. The resulting intramolecular hydrogen bonds mean that more energy is needed to detach a hydrogen atom than in the case of free hydroxyl groups not bonded to hydrogen. As a result, the antioxidant properties decrease. In this particular case, the antioxidant properties of myricetin become comparable or lower than those of quercetin, depending on the method of assessing antioxidant activity.

The participation of intramolecular hydrogen bonds in the stabilization of the phenoxyl radical is equally likely for myricetin in the myricetin/kaempferol system and for quercetin in the kaempferol/quercetin system. Nevertheless, these compounds differ in their ability to form intramolecular hydrogen bonds. In addition, the differences in the antioxidant activity of the compounds in the indicated pairs may be significant enough to mask the stabilizing effect of the emerging intramolecular hydrogen interactions. The validity of this conclusion seems to be confirmed by the data obtained by the DPPH method for the pair myricetin/quercetin at the lowest concentration levels. According to these, as a reminder, myricetin has a lower antioxidant effect than quercetin. This observation may also be explained by the lower accuracy of the DPPH method. In addition, and more importantly, the unpaired electron on the DPPH radical is less available to myricetin (due to its structure), resulting in less reactivity of this compound, similar to the formation of intramolecular hydrogen bonds, and finally worsening the antioxidant effect [23,24].

### 3.2. Resultant Antioxidant Effects in Mixtures

As regards the comparison of the experimentally estimated antioxidant properties of binary mixtures of antioxidants with their properties calculated from the data obtained for individual components (these data in the plots are shown respectively by a solid line with "x" and a dashed line with triangles), which is important for this study, at first glance it seems that the courses of the experimental and calculated curves are similar. This fact suggests the additive antioxidant effect. However, the statistical analysis of the obtained results revealing the significance of the difference (see the Fischer coefficient values in Table 2 and Table 3) confirms the lack of differences between the curves only for 21 out of 54 of all tested systems. Thus, the additive effect occurs in a minority (only in 38% of the systems studied). This effect is more common in the DPPH method (12 cases compared to 9 cases observed in the ABTS method). Regardless of the method, the additive properties of the ingredients were observed only for the kaempferol/quercetin pair for all tested volume ratios at the concentrations of 0.1 and 0.2 mmol/L. Thus, these data expand the set of information about the possible additive effect of quercetin not only in combination with ascorbic acid and alpha-tocopherol, but also with kaempferol [25].

The obtained experimental data generally indicate the antagonistic effect of the tested antioxidants in the binary mixtures, while in the literature on the subject, antagonism was demonstrated only for quercetin in combination with caffeic acid. The validity of the superiority of the antagonistic effect over the expected additive and/or synergistic effect in a mixture of compounds is confirmed by the data [18]. However, according to the results presented here, the magnitude of this effect depends on the method used to evaluate the antioxidant properties (generally, the DPPH method has higher Fischer coefficient values). In addition, it depends on the type of antioxidant (its structure), its volume used in the measurement system and its concentrations. In this context, antagonistic properties are particularly characteristic for systems with myricetin, with high F values obtained for concentrations of 0.03 and 0.1 mmol/L, regardless of the volume ratio and the type of second antioxidant. It is worth noting that for the concentration of 0.2 mmol/L and the myricetin/kaempferol pair, either an additive or a synergistic effect is observed in the ABTS method, depending on the ratio (see Figure 2 and Table 2). Considering the earlier discussion on the susceptibility of the myricetin molecule to the formation of intramolecular hydrogen bonds and their involvement in the reduction of the antioxidant effect of the solution of this compound, it is highly probable that these bonds are also responsible for the changes in the resultant activity of the mixture. To the best of our knowledge, this is the first report showing a concentration-dependent change in the resultant antioxidant effect of the same flavonoids mixture and perhaps revealing the reason for the divergent results in the literature, bearing in mind that most often this type of research is carried out for single concentrations of compounds. Nevertheless, the synergism shown in this paper for the myricetin/kaempferol pair is confirmed by the research of Hidalgo [18]. 

The non-additive antioxidant effect of ingredients in mixtures of various antioxidants is the subject of many papers [25,26,27]. Their authors indicate many possible reasons for this behavior of compounds. Not wanting to duplicate information, a summary of the known causes of antagonistic and synergistic antioxidant effects is available in [13,16]. In the context of flavonoids, specifically the antioxidant effect of myricetin and quercetin in their binary mixtures with glutathione, phenolic acids, curcumin and catechin, which was the subject of research presented in [28,29,30,31], the authors of these papers explain the antagonism by the formation of complexes between antioxidants and/or auto-oxidation of antioxidants. In the latter case, myricetin with pyrogallol configuration in one of the rings can undergo auto-oxidation and form a superoxide radical (O_2_^•−^), which leads to a weakening of its antioxidant effect.

The results presented in this paper, showing comparable or lower effects of the myricetin solution (a stronger antioxidant) compared to the quercetin solution (a weaker antioxidant) and antagonism in their mixture, proved the participation of intramolecular hydrogen bonds formed between the hydroxyl groups of the antioxidant molecule in reducing the mixture’s antioxidant activity, as suggested in the literature [32,33]. In other words, when the -OH groups are involved in the formation of hydrogen bonds (see Figure 4), it makes it difficult to detach the proton from the phenolic group, which is manifested by the lack of influence of these groups on the antioxidant properties (see Figure 2) or by a decrease in antioxidant activity (see Figure 3), depending on the research method used. Considering that the ABTS^•+^/DPPH^•^ radical neutralization process is based on the single electron transfer (SET) mechanism accompanied by the proton detachment from the phenolic -OH group, in general, the stronger the hydrogen bond, the weaker the antioxidant activity of the hydrogen atom/proton-donating group in the antioxidant molecule and the lower the resultant effect of the mixture.

## 4. Materials and Methods

### 4.1. Chemicals

Kaempferol, myricetin, quercetin, 2,2′-azinobis(3-ethylbenzothiazoline-6-sulfonic acid) diammonium salt (ABTS) and 2,2′-diphenylpicrylhydrazyl (DPPH) were purchased from Sigma Aldrich (Poznań, Poland). Methanol (MeOH) and potassium persulfate (di-potassium peroxdisulfate) were purchased from the Polish Chemical Plant POCh (Gliwice, Poland). Water was purified on a Milli-Q system from Millipore (Millipore, Bedford, MA, USA).

### 4.2. Measurements of Antioxidant Properties

Measurements of antioxidant properties of the examined antioxidants and their binary mixtures were assessed using two spectrophotometric methods presented below in which changes of the colored radical cations, i.e., ABTS^•+^ and DPPH^•^, were monitored. All measurements were performed using a UV Probe-1800 Spectrophotometer (Shimadzu, Kyoto, Japan) and an optical glass cuvette (1 cm × 1 cm × 3.5 cm). The studies used methanol solutions of kaempferol, myricetin and quercetin prepared at three concentration levels, i.e., 0.03, 0.1 and 0.2 mmol/L, within the range in which these compounds occur in plants. The volume ratios of antioxidant standard solutions and antioxidant standard solutions in binary mixtures are presented in Table 1.

#### 4.2.1. ABTS Method

ABTS cation radical absorbance changes were monitored at 744 nm. The ABTS cation radical (ABTS^●+^) was formed by the reaction of 2,’-azinobis (3-ethylbenzenothiazoline-6-sulfonate) (ABTS) with potassium persulfate after mixing 5 mL of 7 mmol/L ABTS solution with 88 µL of 140 mmol/L potassium persulfate. Then, the mixture was incubated in the dark for 16 h and diluted with methanol until the absorbance value equaled 0.7 ± 0.05 [34]. During measurements, 2900 µL of methanolic solution of ABTS^●+^ was mixed in a 4 mL test tube with 100 µL of antioxidant solution or antioxidant binary mixture. The exact compositions of the examined measurement systems are shown in Table 1. Pure methanol was used to zero the spectrophotometer.

After an hour-long reaction, the changes in the radical absorbance were expressed as inhibition percent (% I), which is a measure of the antioxidant properties of the tested antioxidants and their two-component mixtures. The % I values were calculated according to the following equation:

(1) $$I\left(\%\right)=(1-\frac{A_{60}}{A_{0}})\cdot 100\%$$

where A_0_ and A_60_ are the values of ABTS^●+^ absorbance at 0 and 60 min of the radical neutralization reaction, respectively.

#### 4.2.2. DPPH Method

The concentration of the DPPH radicals after their reaction with the examined antioxidant or antioxidant binary mixture was estimated by the slightly modified Brand–Williams method [35]. A DPPH^•^ methanolic solution of initial absorbance 0.7 ± 0.05 at 516 nm (2900 μL) was mixed with a methanolic solution (100 µL) of the examined substance or antioxidant binary mixture in a 4 mL test tube (see Table 1). The mixture, after vigorous shaking for 30 s, was transferred into an optical glass cuvette (1 cm × 1 cm × 3.5 cm), which was immediately placed in a spectrophotometer. The absorbance decrease at 516 nm was monitored continuously for 60 min. Methanol was used to zero the spectrophotometer.

The % I values were calculated according to the above equation (see description of ABTS method) only with the difference that A_0_ and A_60_ were the values of DPPH^•^ absorbance at 0 and 60 min of the radical neutralization reaction, respectively.

### 4.3. Statistical Analysis

All experimental data are presented as mean values of five independent measurements ± standard deviation (SD). The one-way analysis of variance (ANOVA) and Fisher coefficient (*F*) value were used to assess the influence of experimental factors on the activity. If the calculated value of *F* (*F_cal_*) exceeds the tabular value *F* (*F_tab_*), this indicates a statistically significant influence of the given parameter. To determine the significance of each Fisher coefficient, the *p*-values were used. The values were considered to be significantly different when the results of the compared parameters differed at the *p* = 0.05 significance level. The statistical analysis was performed using Excel (Microsoft Excel 2010).

## 5. Conclusions

The presented paper shows and discusses the antioxidant properties of selected flavonoids (myricetin, quercetin and kaempferol) and their binary mixtures, proving the following:

- The dominant resultant effect in the mixtures is antioxidant antagonism;
- The magnitude of the observed antagonism depends on the mutual relationships of individual components, their concentrations and the method used to assess antioxidant properties, and, more importantly;
- The observed effect results from the formation of intramolecular hydrogen bonds between phenolic groups of the antioxidant molecule.

The latter conclusion complements and extends the knowledge about the non-additive antioxidant properties of compounds in mixtures. In order to correctly predict the antioxidant behavior of a mixture of natural polyphenols, it is necessary to take into account the impact of their interactions. The more so, as it has been shown, as confirmed by literature data [27], that only compounds that are non-hydrogen-bonded (free) possess activity (electron transfer mechanism) and that the rate of their reaction depends on the strength of the hydrogen bond.

Detailed knowledge of the antagonistic, synergistic and additive antioxidant effects of various antioxidant blends can be helpful in the proper design of functional foods and supplements. Nevertheless, given the complexity of the issue, further experiments are needed to expand our understanding of the antioxidant activity of mixtures.

## Figures and Tables

**Table 1:** Volumes of myricetin, kaempferol and quercetin solutions used for the determination of the antioxidant properties of these compounds and their binary mixtures.

Components Volumes in μL	System Number																	
	1	2	3	4	5	6	7	8	9	10	11	12	13	14	15	16	17	18
Myricetin in MeOH	20	50	80	-	-	-	-	-	-	20	50	80	-	-	-	20	50	80
Kaempferol in MeOH	-	-	-	20	50	80	-	-	-	80	50	20	20	50	80	-	-	-
Quercetin in MeOH	-	-	-	-	-	-	20	50	80	-	-	-	80	50	20	80	50	20
MeOH	80	50	20	80	50	20	80	50	20	-	-	-	-	-	-	-	-	-
Total volume	100																	

**Table 2:** Statistical significance (*F* and *p* values) of the difference between the experimental (I_e_) and calculated (I_c_) antioxidant activity expressed as the inhibition percent (I) determined by the ABTS method for the individual binary mixtures of the tested compounds for different volume ratios at three concentration levels, together with the resultant value of the difference (I_e_-I_c_) and the resultant antioxidant effect of the ingredients in the mixture (observed effect).

Compound Pairs	Concentration (mmol/L)	Volume Ratios (v/v)	ABTS		(I_e_-I_c_)-Values	Observed Effect
			F-Values	*p*-Values		
Myricetin/Kaempferol	0.03	20/80	32.49	0.0046	negative	antagonism
		50/50	55.51	0.0017	negative	antagonism
		80/20	15.35	0.0173	negative	antagonism
	0.1	20/80	19.02	0.0121	negative	antagonism
		50/50	13.03	0.0225	negative	antagonism
		80/20	12.55	0.0239	negative	antagonism
	0.2	20/80	0.09	0.7716	negative	antagonism
		50/50	23.76	0.0082	positive	synergism
		80/20	0	1	-	additivism
Kaempferol/Quercetin	0.03	20/80	11.28	0.0283	negative	antagonism
		50/50	20.67	0.0104	negative	antagonism
		80/20	28.22	0.0060	negative	antagonism
	0.1	20/80	0.04	0.5700	-	additivism
		50/50	6.95	0.0577	-	additivism
		80/20	2.32	0.2017	-	additivism
	0.2	20/80	0.28	0.6246	-	additivism
		50/50	5.99	0.0706	-	additivism
		80/20	2.06	0.2244	-	additivism
Myricetin/Quercetin	0.03	20/80	38.06	0.0035	negative	antagonism
		50/50	58.19	0.0016	negative	antagonism
		80/20	35.90	0.0039	negative	antagonism
	0.1	20/80	17.06	0.0144	negative	antagonism
		50/50	33.70	0.0040	negative	antagonism
		80/20	20.33	0.0110	negative	antagonism
	0.2	20/80	8.03	0.0472	negative	antagonism
		50/50	20.48	0.0106	negative	antagonism
		80/20	7.24	0.0546	-	additivism

**Table 3:** Statistical significance (*F* and *p* values) of the difference between the experimental (I_e_) and calculated (I_c_) antioxidant activity expressed as the inhibition percent (I) determined by the DPPH method for the individual binary mixtures of the tested compounds for different volume ratios at three concentration levels, together with the resultant value of the difference (I_e_-I_c_) and the resultant antioxidant effect of the ingredients in the mixture (observed effect).

Compound Pairs	Concentration (mmol/L)	Volume Ratios [v/v]	DPPH		(I_e_-I_c_)-Values	Observed Effect
			F-Values	*p*-Values		
Myricetin/Kaempferol	0.03	20/80	162.79	0.0002	negative	antagonism
		50/50	159.36	0.0002	negative	antagonism
		80/20	16.27	0.0157	negative	antagonism
	0.1	20/80	10.56	0.0314	negative	antagonism
		50/50	8.36	0.0445	negative	antagonism
		80/20	19.88	0.0112	negative	antagonism
	0.2	20/80	18.83	0.0261	negative	antagonism
		50/50	54.95	0.0021	negative	antagonism
		80/20	20.31	0.0011	negative	antagonism
Kaempferol/Quercetin	0.03	20/80	11.89	0.0985	negative	antagonism
		50/50	49.84	0.0021	negative	antagonism
		80/20	69.86	0.0011	negative	antagonism
	0.1	20/80	4.59	0.0985	-	additivism
		50/50	4.83	0.0927	-	additivism
		80/20	7.05	0.0566	-	additivism
	0.2	20/80	2.75	0.1723	-	additivism
		50/50	2.74	0.1732	-	additivism
		80/20	6.01	0.0018	-	additivism
Myricetin/Quercetin	0.03	20/80	72.05	0.0011	negative	antagonism
		50/50	101.02	0.0006	negative	antagonism
		80/20	38.95	0.0034	negative	antagonism
	0.1	20/80	5.91	0.0717	-	additivism
		50/50	0.97	0.3790	-	additivism
		80/20	3.29	0.144	-	additivism
	0.2	20/80	2.93	0.1620	-	additivism
		50/50	0.41	0.5571	-	additivism
		80/20	0.81	0.4187	-	additivism

## Data Availability

Not applicable.
